# The Response of Neotropical Dragonflies (Insecta: Odonata) to Local and Regional Abiotic Factors in Small Streams of the Amazon

**DOI:** 10.3390/insects10120446

**Published:** 2019-12-12

**Authors:** José Max Barbosa Oliveira-Junior, Karina Dias-Silva, Maria Alexandra Teodósio, Leandro Juen

**Affiliations:** 1 Programa de Pós-Graduação em Zoologia (PPGZOOL), Programa de Pós-Graduação em Ecologia (PPGECO), Laboratório de Ecologia e Conservação (LABECO), Universidade Federal do Pará (UFPA), Rua Augusto Correia, N.1, Bairro Guamá, Belém 66075-110, Pará, Brazil; diassilvakarina@gmail.com (K.D.-S.); leandrojuen@ufpa.br (L.J.); 2 Instituto de Ciências e Tecnologia das Águas (ICTA), Universidade Federal do Oeste do Pará (UFOPA), Rua Vera Paz, s/n (Unidade Tapajós) Bairro Salé, Santarém 68040-255, Pará, Brazil; 3 Centro de Ciências do Mar (CCMAR), Universidade do Algarve (UAlg), Campus de Gambelas, 8005-139 Faro, Portugal; mchichar@ualg.pt; 4 Programa de Pós-Graduação em Biodiversidade e Conservação (PPGBC), Universidade Federal do Pará (UFPA), Rua Coronel José Porfírio, N. 2515, Bairro São Sebastião, Altamira 68372-040, Pará, Brazil

**Keywords:** Anisoptera, bioclimatic variables, forest cover, landscape, rainforest streams, variance partitioning, Zygoptera

## Abstract

Since the relative role of local and regional abiotic factors on the Odonata diversity in rainforest streams is still poorly understood, we evaluated the effects of these factors on adult Odonata (Insecta) from preserved and altered streams in the Amazonian region. Adult Odonata were sampled in 98 streams in the Eastern Amazon, Pará, Brazil. Six variables were used to measure local environmental factors: habitat integrity index; mean canopy over the channel; and four physical and chemical descriptors of the water. To measure regional environmental factors, six variables were also used: altitude gradient, three bioclimatic variables and two percentage forest variables. In partial redundancy analysis, both abiotic factors (local and regional) were important to explain the variation in the Odonata community. The Odonata community can be influenced by regional and local factors. The relationship between Odonata and the local (e.g., integrity, canopy cover, and physical and chemical descriptors of the water) and regional (e.g., bioclimatic and forest cover variables) environmental variables recorded in this study has important implications for the use of these organisms to monitor small streams of the Eastern Amazon. The scale at which habitat is measured is an important issue in community structuring studies considering the rapid environmental changes. It is of great importance to consider the different scales in studies assessing community structure, once an adequate habitat must meet the ecological needs of all stages of the life of the Odonata.

## 1. Introduction

Linking geographic variation of animal communities with variation in local and regional abiotic variables is a common objective in community ecology studies [1,2]. Understanding the structure of these associations in the tropical region is extremely important because of the great biodiversity, now facing increasingly frequent environmental changes [3,4]. Therefore, understanding the primary factors responsible for determining community structure and species distribution has become one of the main objectives in ecology [5] under the conservationist perspective [4]. In tropical regions, local diversity can be regulated by local and regional factors [6]. Many papers have addressed the effects of local factors on diversity [7,8] or interactions such as competition, predation, and parasitism (e.g., [9,10]). Still, in recent years, factors that occur in larger scales have also been identified determinant for the richness of local communities [11].

The properties of a site are often a consequence of external processes or dynamics, such as migration and landscape effects. The migration of multiple species among sites connects neighboring communities along environmental gradients, causing variations in the metacommunity structure established in the region [12]. The new habitats in a landscape (regional scale) are colonized by individuals from the regional pool of species that maintain the local populations through dispersal. The establishment of organisms, populations, and communities depends on local environmental conditions and interactions with previously established species [13].

There is a long history of studies in aquatic environments that have assessed the relationship between biodiversity and environmental gradients for aquatic macroinvertebrates [14,15] and fish [16]. Macroinvertebrates can be influenced by both local (e.g., substrate and physical-chemical characteristics of the water), and regional factors (e.g., climate, altitude and latitude variation; [17]). Therefore, species composition may vary depending on the environmental characteristics of each system, or following geographical distances and dispersal limits of the landscape [18]. In addition, the patterns associated with local and regional factors may be altered by changes in the land use of areas surrounding aquatic systems, causing biodiversity loss [14]. Any change in local or regional factors may affect the composition and distribution of aquatic organisms [19].

The distribution, richness, and composition of the Odonata (Insecta) are known for being closely associated with changes in environmental resources [20]. Odonata have relatively long lifespans in comparison to many other insects (e.g., most Dipterans) [21]; up to one year in the tropics [22]. They are widely distributed in aquatic systems [23,24], and present a dual life cycle, where larvae are aquatic and adults are terrestrial [25]. The fact that Odonata larvae and adults occupy two different environments suggests that this group can provide valuable information on changes occurring in both aquatic and terrestrial environments [26].

Studies have shown the importance of environmental gradient effects on the functional traits of macroinvertebrates (e.g., [27,28,29]). Environmental filters can select the species that will occur in a given location due to limiting environmental conditions such as light, temperature, and humidity [30]. This approach of environmental filters [31,32] has been followed in studies on freshwater macroinvertebrate assemblages [33,34]. These studies have generally considered multiple nested scales, with the assumption that factors at different scales are hierarchically structured, and that factors at larger scales determine, at least in part, those at smaller scales [35,36,37]. Still, the relative role of landscape and local environmental variables on the diversity of Odonata in tropical streams are poorly understood, possibly because previous studies did not use large enough scales [15].

In this context, our objective was to evaluate the effects of abiotic factors to local (e.g., quantitative physical variables of the stream) and regional scales (e.g., altitude, bioclimatic variables, and forest cover) on Odonata adult communities in preserved and altered streams in the Eastern Amazon. We hypothesize that both sets of abiotic factors structure the adult community of Odonata in the Eastern Amazon. Whereas, different studies have reported the association of Odonata species or groups with local abiotic factors (e.g., [38,39]), climate (e.g., [40]), and surrounding land use conditions (e.g., [41,42]). We expect the Zygoptera community to be structured by local/regional factors in greater intensity than the community of Anisoptera. The low dispersal capacity of species with smaller body sizes, such as the Zygoptera species [43], should increase dependence on environment conditions (e.g., physical and chemical variables, river channel and vegetation structure) and, consequently, greater habitat specialization [44]. In contrast, the high vagility of Anisoptera species (individuals of larger body size), provides species with the ability to leave environments with unfavorable environmental conditions.

## 2. Materials and Methods

### 2.1. Study Areas

**Figure 1:**
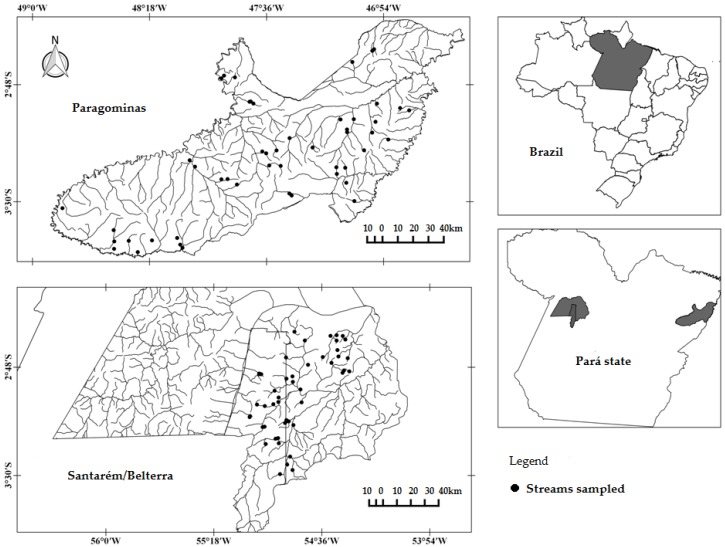
Drainage network and streams sampled in the regions of Santarém/Belterra and Paragominas, Eastern Amazon, Pará, Brazil. (Source: map developed using the software ArcGIS [47].

Two regions of the Eastern Amazon region were prospected, being one in the municipality of Paragominas, and the other in the municipalities of Santarém/Belterra, Pará, Brazil. The municipality of Paragominas (1.9 Mha) is located in the northeast of the state of Pará (02°59′51″ S, 47°21′13″ W). The mean annual precipitation is 1,766 mm/year, the mean annual temperature is 27 °C and the relative humidity is 81% [45]. The Santarém/Belterra municipalities (1 Mha; 02°26′22″ S, 54°41′55″ W and 02°41′54″ S, 54°53′18″ W, respectively) are located in the western region of the state of Pará, with a slightly higher rainfall (on average 1920 mm/year), mean annual temperature of 25 °C and relative humidity of 86% [46]. The prevailing climate of Paragominas is “Af”, of Santarém is “Am”, and of Belterra is “Amw” according to the Köppen classification, and are characterized as tropical rainy with a short well-defined dry season (Figure 1). The shortest distance between sites of the two areas was 1100 km and the largest was 2210 km, which ensures a large, regional-scale, variation of environmental variables.

**Figure 2:**
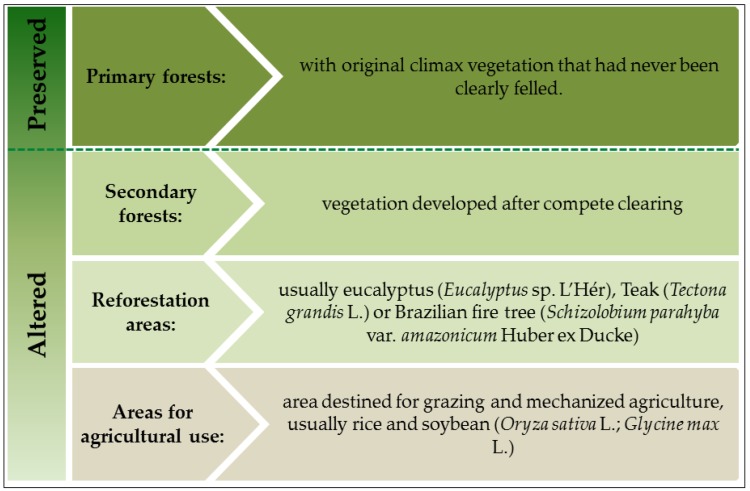
Land use gradient (from agriculture to secondary forest; that demonstrates altered environments) and primary forests (that demonstrates preserved environments) in the regions of Santarém/Belterra and Paragominas municipalities, Eastern Amazon, Pará, Brazil.

The vegetation of the Paragominas region is a dense ombrophylous forest [45], and of Santarém and Belterra is a tropical rainforest with limited areas of Amazonian savannas located in the northwestern region [45]. Both regions exhibit a land use gradient [45,48,49] encompassing preserved and altered areas (Figure 2).

### 2.2. Data Sampling

#### 2.2.1. Biological Sampling and Laboratory Procedures

A total of 98 streams (streams ranging from first to third order, an average of 2 to 5 m wide) were sampled in the two regions during the dry season; 50 were in Paragominas (from June to August 2011) and 48 in the region of Santarém and Belterra (from July to August 2010). The rainy season (December to May) was not included in the study because of the ecophysiological requirements of Odonata (high precipitation may reduce the effectiveness of sampling procedures; see [23,50,51]. In addition, some studies have also shown that the greatest richness and abundance of adult Odonata occur in the dry season [49,52,53]. The shallower depth of the water column during this period causes these insects to focus on smaller areas, which allowed us to find and capture them more easily [54,55]. The focus on a single seasonal period also reduces sampling “noise” in the analyses and results [56].

**Figure 3:**
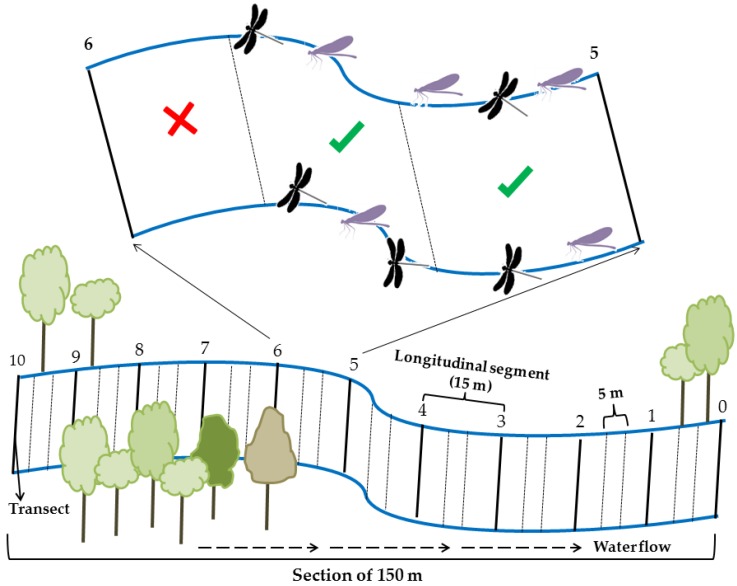
Schematic drawing of the division into segments of the 150-m stretch for Odonata sampling in streams in two regions (Santarém/Belterra and Paragominas municipalities), Eastern Amazon, Pará, Brazil.

A 150 m reach was delimited in each stream. Each reach was subdivided into 10 longitudinal sections of 15 m each, separated by transects margin to margin (see [45]). The longitudinal sections of 15 m were subdivided into three segments of five meters each, and only the first two segments from each section were sampled, amounting to 20 segments of 5 m for each stream (Figure 3).

Air temperature and humidity were measured in a shaded location near each stream. The samples were collected between 10:00 a.m. and 2:00 p.m., when the sun rays reached the stream, minimum conditions to ensure that the different adult Odonata groups (thermal conformers, heliotherms, and endotherms) were active at the collection time [50,51,57]. The sighted adult dragonflies were collected using a butterfly net (40 cm Ø) following the collection protocol used in Oliveira-Junior et al. [49] and conditioned following Lencioni [58].

Taxonomic keys and illustrated guides were used to identify the specimens [58,59,60,61,62,63,64]. The specimens identified were compared with identified material from the collection of the Museum of Zoology of the Federal University of Pará, Brazil, and then were stored as voucher material.

#### 2.2.2. Local Abiotic Factors

In total, six variables were used to measure local environmental factors: habitat integrity index (HII); mean canopy over the channel; and four physical and chemical descriptors of the water (see Table S1).

The HII [65] was used to quantify the integrity of each stream and classify it in preserved and altered streams. This index has 12 items that assess the environmental conditions of streams, visually evaluating the following characteristics: land use pattern adjacent to the riparian vegetation; width of the riparian forest and its preservation state; state of the riparian forest within a 10 m area around the stream; condition of the channel as to the type of sediment and presence of retention devices; structure and wear of stream margins; and river bed characteristics (substrate, aquatic vegetation, debris, and disposition of rapids, pools, and meander areas). Each item is made up of four to six alternatives. These alternatives are ranked in an increasing integrity order, and the index value ranges from 0 (lowest integrity) to 1 (highest integrity). This index is directly related to the degree of environmental conservation and has been successfully used in other studies to evaluate aquatic system integrity [41,66,67,68].

Mean channel shading was estimated using a convex densitometer at the central point of the channel, where four measurements were taken, upstream, downstream, left, and right banks. Canopy cover has often been reported as one of the main physical characteristics of the habitat that influences the distribution pattern of Odonata in tropical streams [39,42].

In addition, four physical and chemical descriptors of the water were measured in each stream using a U-51 model Horiba^®^ multiparameter probe: water temperature (°C); electrical conductivity (μS/cm), dissolved oxygen (mg/L), and pH. Several studies have demonstrated the importance of physicochemical variables of the watercourse for structuring aquatic insect communities, such as water temperature, electrical conductivity [39,69], dissolved oxygen [70], and pH [71].

#### 2.2.3. Regional Abiotic Factors (Bioclimatic and Soil Cover Variables)

In total, six variables were used to measure regional/landscape environmental factors: altitude gradient, three bioclimatic variables and two forest percentage variables (see Table S1).

The altitude gradient and three bioclimatic variables used ((1)—annual mean temperature; (2)—annual precipitation; and (3)—coefficient of seasonal rainfall variation) were obtained from the WorldClim database, version 1.4. The resolution used was approximately 1 km at the equator (30 arc-seconds). WorldClim provides 19 bioclimatic variables (see http://www.worldclim.org/; [72]) widely used in research studies.

Altitude has been significantly related to the taxonomic variability of macroinvertebrates in the tropics [73]. Bioclimatic variables have often been used in studies using species distribution models [72,74,75] and can influence the characteristics of the landscape and as well as community dynamics [74]. For example, the annual mean temperature can potentially influence the dynamics of aquatic populations [74,76]; annual precipitation is of great importance in structuring macroinvertebrate communities in tropical streams [74,77]; and coefficient of seasonal rainfall variation, can be considered an indirect measure of environmental variability in the system [74].

Land cover data were obtained by interpreting LandSat images, made available by the environmental organization named Amazonian Institute of Man and Environment (IMAZON). Forest cover was defined by 200 m buffers, delimiting the landscape where the percentage of coverage was estimated. The proportion of habitats in preserved environments or of natural cover is among the main variables that explain species distribution and community structure in natural environments [39,42,78].

### 2.3. Data Analysis

To evaluate the distinction between the conservation categories of the streams, the values of the 12 items of the HII that describe the prevailing environmental conditions of the study streams were summarized in a principal component analysis (PCA). To determine which principal components should be retained for analysis, we used the randomness obtained by the broken-stick model [79]. To test whether the conservation categories (preserved and altered) were significantly different from one another, the scores generated by the PCA were tested using Student’s *t*-test (*p* < 0.05).

To test differences in abundance and species richness based on conservation categories of streams, we used inference based on the confidence interval of 95%, in which the groups were considered different when the confidence intervals did not overlap between groups [80].

A principal coordinates analysis (PCoA) was used to summarize the species composition in the environments according to the conservation category (preserved and altered), using the Bray-Curtis dissimilarity index [81]. A multivariate analysis of permutational variance (PERMANOVA; pseudo-F) was performed with 9999 replications [82] to test for significant differences in species composition among conservation categories. The PERMANOVA does not assume normality or homoscedasticity and allows to test the interactions between the factors. However, significant results obtained using the PERMANOVA may indicate differences due to between-group dissimilarity as well as to the variation of within-group dispersion. The potential role of each of these factors was evaluated through a permutational dispersion multivariate analysis (PERMDISP; *P*_perm_), using the distance from each sample to the mean of the group [83], also using the Bray–Curtis distance.

A partial redundancy analysis (pRDA) [81] with the matrices of environmental variables (local and regional) and of species composition (Odonata, Anisoptera, and Zygoptera) was conducted to evaluate the relative importance of local and regional factors on the Odonata community. The variation in community structure was partitioned in the following fractions: [LO] pure local environmental factors (variance explained only by local environmental variables, and not shared with regional variables); [RE] pure regional environmental factors (variance explained only by the landscape variables, regardless of local environmental variables); [RE:LO] a fraction of variation shared by regional and local variables; and [RS] a fraction or residue unexplained by local or regional environmental variables [84]. The significance of each fraction was tested by permutation tests using 999 randomizations [85]. All environmental variables, except pH, were Z-transformed, homogenizing the scales of the different variables [86].

All analyses (with biotic data) were performed considering the order data as a whole (that is, total Odonata) and by suborder separately (that is, Anisoptera and Zygoptera). All analyses were performed with routines of the R software [87], using the vegan, Varpart, labdsv, and mgcv packages.

## 3. Results

### 3.1. Conservation Categories of Streams

**Figure 4:**
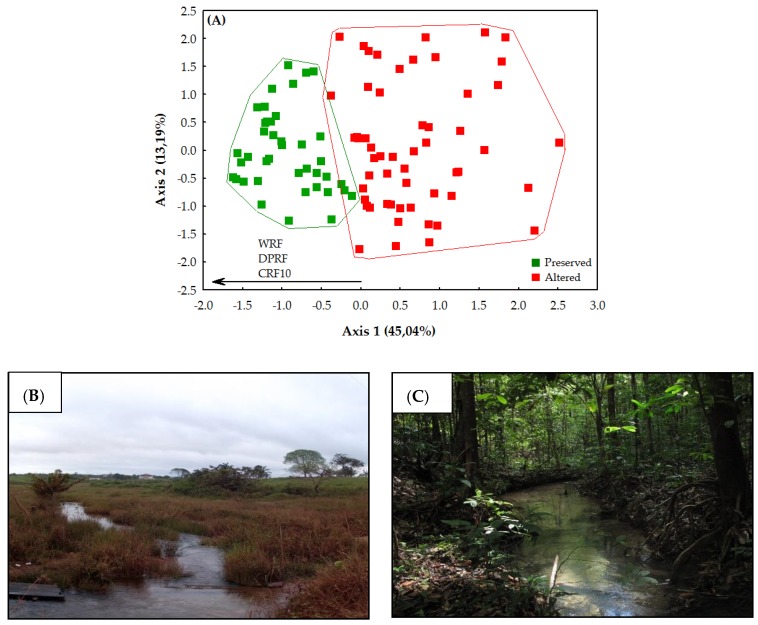
(**A**) Ordination of environmental variables (values of 12 items of habitat integrity index (HII)); and example of stream conservation category (**B**) altered and (**C**) preserved in two regions of Eastern Amazon, Pará, Brazil. (WRF = width of the riparian forest; DPRF—degree of preservation of the riparian forest; CRF10—condition of the riparian forest within a radius of 10 m).

The HII values varied from 0.15 to 0.99. Based on this variation, the 98 streams were classified in two arbitrary categories of conservation (Figure 4): altered (HII = 0.15–0.69; 56 streams; Figure 4B) and preserved (0.70–0.99; 42 streams; Figure 4C). The separation of the streams into two conservation categories was significant (t = 13.292; df = 96; *p* < 0.001).

The association of the two PCA axes represented 58.23% of the environmental variation. Only the first axis was analyzed, given that the second axis did not present an observed value greater than that estimated by the broken-stick procedure (which was adopted whenever a situation of this type arose). The first axis explained 45.04% of the results (eigenvalue = 5.40). In this analysis, the samples were separated by the conservation category. The preserved streams had a positive relationship with environmental integrity, being grouped in the direction of the highest values for the width and degree of preservation of the riparian forest (Figure 4A). The impacted streams were characterized by a significant loss and changes in the state of preservation of the riparian forest, with a group of these streams being associated negatively with the integrity of this vegetation (Figure 4A).

It is important to note here that the variables that most contributed to the formation of the first axis are closely related to the physical structure of the riparian vegetation. These variables are associated negatively with the level of conservation of these environments, including the width of the riparian forest (WRF), degree of preservation of the riparian forest (DPRF), and the condition of the riparian forest within a radius of 10 m (CRF10) (Figure 4A).

### 3.2. Abundance, Species Richness and Composition of Odonata

**Figure 5:**
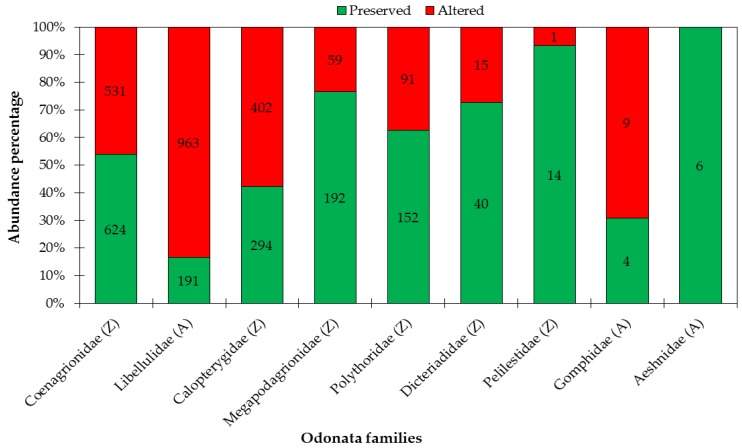
Abundance of Odonata families (Zygoptera-Z and Anisoptera-A), per stream conservation category (preserved and altered) sampled in two regions of Eastern Amazon, Pará, Brazil. Bars represent the percentage of abundance (according to Y axis), and numbers within bars represent absolute abundance.

A total of 3588 adult Odonata specimens were collected, encompassing nine families, 49 genera, and 134 species. The suborder Zygoptera was represented by 2415 individuals, distributed among six families (Calopterygidae, Coenagrionidae, Dicteriadidae, Megapodagrionidae, Perilestidae, and Polythoridae), 20 genera, and 71 species. Coenagrionidae was the most abundant Zygoptera family (*n* = 1155), and more than 50% of this total (*n* = 624) is in preserved streams (Table S2). Anisoptera was represented by 1173 individuals within three families (Aeshnidae, Gomphidae, and Libellulidae), 29 genera, and 63 species. Libellulidae was the most abundant Anisoptera family (*n* = 1154), and 83% of this total (*n* = 963) is in altered streams (Figure 5; see Table S2, for an overview of all sampled species).

**Figure 6:**
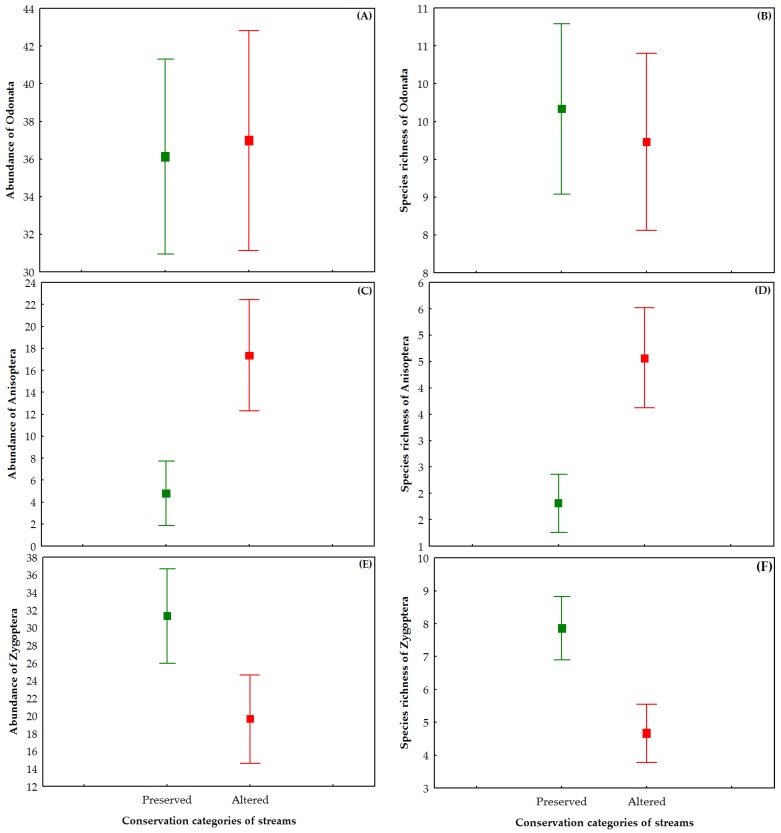
Abundance and species richness (average ± confidence interval 95%) by site conservation status: (**A**) abundance Odonata; (**B**) species richness Odonata; (**C**) abundance Anisoptera; (**D**) species richness Anisoptera; (**E**) abundance Zygoptera; and (**F**) species richness Zygoptera.

No differences in abundance and richness of Odonata (Anisoptera and Zygoptera) species were observed between the conservation categories of streams (Figure 6A,B). Considering the results for each suborder, Anisoptera had the highest abundance and richness in altered sites, which averaged 13 individuals and three species more than the preserved sites (Figure 6C,D). For Zygoptera, abundance and richness were higher in preserved sites, which averaged 12 individuals and three species more than altered sites (Figure 6E,F).

**Figure 7:**
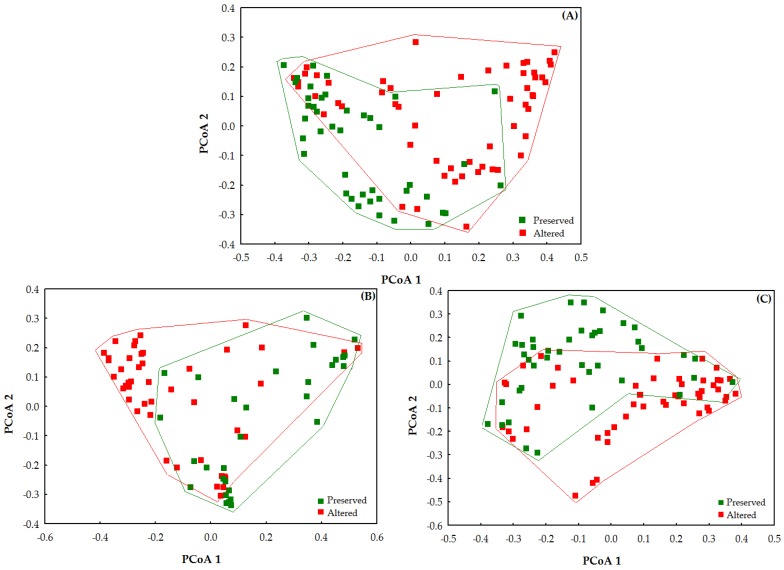
Ordination using coordinate analysis—PCoA for the composition of the following species: (**A**) Odonata; (**B**) Anisoptera, and (**C**) Zygoptera grouped per stream conservation category (preserved and altered) sampled in two regions of Eastern Amazon, Pará, Brazil.

The Odonata species composition (considering Anisoptera and Zygoptera together) differed significantly between the two conservation categories (pseudo-F = 10.323; *p* = 0.001) (Figure 7A). The same pattern was observed separately for Anisoptera (pseudo-F = 7.110; *p* = 0.001; Figure 7B) and for Zygoptera (pseudo-F = 7.937; *p* = 0.001) (Figure 7C). The species composition also differed between groups, as shown by the difference in heterogeneity recorded for Odonata and for Zygoptera (F = 8.029; *P*_perm_ = 0.012; F = 3.115; *P*_perm_ = 0.001 respectively). Odonata composition was more heterogenous in altered than preserved sites, while the opposite pattern was recorded for the Zygoptera. No difference was recorded for Anisoptera (F = 3.524; *P*_perm_ = 0.103).

### 3.3. Response of Odonata Communities to Local and Regional Abiotic Factors

Variance partitioning showed that the effects of local and regional abiotic factors on the composition of dragonfly species differed (Table 1). The component shared by the two sets of abiotic variables [RE:LO] played an important role in determining the variation of Odonata species composition (total, Anisoptera and Zygoptera) (*R*^2^_adj._ = 0.24), followed by the pure local component [LO] (*R*^2^_adj._ = 0.12; *p* < 0.001), and finally, by the pure regional abiotic component [RE] (*R*^2^_adj._ = 0.07; *p* < 0.001) (Table 1). The component shared by the regional and local abiotic factors [RE:LO] also played an important role in determining the composition of Anisoptera species (*R*^2^_adj._ = 0.15), followed by the pure local abiotic factors [LO] (*R*^2^_adj._ = 0.11; *p* = 0.001), and by the pure regional abiotic factors [RE] (*R*^2^_adj._ = 0.03; *p* = 0.036) (Table 1). The same pattern was observed for Zygoptera ([RE:LO] *R*^2^_adj._ = 0.24; [LO] *R*^2^_adj._ = 0.10; *p* < 0.001; [RE] *R*^2^_adj._ = 0.08; *p* < 0.001) (Table 1).

## 4. Discussion

The structure of the Odonata community (total Odonata, Anisoptera, and Zygoptera) are correlated to the local and regional abiotic factors, corroborating our hypothesis. Additionally, we observed that only the combination of both factors can explain this variability. Thus, the combination of the local and regional factors is key determinants of the richness and abundance of Odonata species in the studied regions.

Many studies on metacommunities have pointed out that a large percentage of variability remains unexplained [88,89,90]. In our results, almost all of the environmental data explained over half of the composition variability for Odonata, Zygoptera, and Anisoptera. This shows the importance of the environmental component in structuring dragonfly communities. Different processes operate at different spatial scales, and processes that operate on small scales can influence large scale patterns [91]. This may explain the importance of both local and regional factors in determining the structure of Odonata communities.

Local environmental factors have already been identified as important for the biotic structure and organization of aquatic communities [15,42,92,93]. For example, the integrity of the streams proved to be an important factor in structuring the two suborders, but with opposite results. Zygoptera species were sensitive to changes in the physical integrity of the environments, presenting higher abundance in preserved streams, while Anisoptera species presented higher abundance in altered streams. This pattern has been a recurrent in studies on Anisopterans [38,41,42,49,67,94]. Another factor corroborating this argument is the fact Zygoptera species have been associated with more pristine environments while Anisoptera species are associated with open or modified environments in most of the studies that evaluated these orders as bioindicators of environmental quality [38,49,67,94].

Riparian vegetation is highly related to stream integrity and has a strong effect on the Odonata community. Riparian vegetation removal has a negative effect on the abundance and richness of Zygoptera species, many of which are highly dependent on areas covered by dense vegetation. Zygoptera species are small and slim bodied and are more subject to overheating and desiccation because of their high surface:volume ratio, making these individuals more sensitive to environmental variations due to ecophysiological limitations [23,95], and consequently, also more restricted to shaded areas [15]. In contrast, the removal of riparian vegetation may increase the abundance of Anisoptera species. This suborder encompasses heliothermic species. Consequently, their abundance is highly dependent on solar radiation [96], necessary for warming and to begin their activities [50,95,97]. As such, Anisoptera species tend to avoid shaded areas [96,98,99]. This was the case of *Erythrodiplax fusca* (Rambur, 1842) and *Orthemis discolor* (Burmeister, 1839) sampled in this study, which normally avoids these areas.

The high local variability of the streams demonstrates the importance of micro and mesoscale processes in the ecological structuring of benthic communities in different mesohabitats [100,101]. The functional structure of benthic invertebrate communities is significantly related to local variables, being the functional variability of stream segments largely dependent on the microhabitat [102].

Many Odonata species require specific habitat characteristics (usually most Zygoptera; [23,103]). The local environmental conditions tend to be more determinant for the distribution of these specialist species, which leads to the inference that species selection is a key process in determining the structure and dynamics of these communities [104]. This corroborates with other studies evaluating the influence of habitat on the characteristics of the communities that Odonata, in which they identified that some species are associated with specific habitat types [49,105].

The absence of vegetation can lead to increased water temperature [106]. Thus, the reduced richness of Zygoptera species may be a reflection of the removal of the marginal vegetation of these habitats, rather than of the water temperature itself [42,49]. Several studies have shown that Odonata diversity is affected at a local scale mainly due to changes in vegetation [42,107], the integrity of the water body [53,108], physical and chemical habitat variables [53,70], and anthropogenic changes [25,39,41,42,49], as a consequence of the ecophysiological demands of the group. For example, electrical conductivity may indicate the presence of inorganic nutrients that stimulate algal and macroinvertebrates growth in Amazonian streams, thereby increasing food for predators, such as Zygoptera larvae [109]. The amount of wood in the riverbeds also influences the richness of Zygoptera species, probably because these species present epiphytic or endophytic oviposition [53].

At regional level, variables such as precipitation and altitude also have been recorded as important in structuring macroinvertebrate communities in tropical streams [77,110]. Precipitation may affect the dynamics and hydrologic patterns of running waters [111,112]. Consequently, the hydrologic dynamics affect the structure of aquatic macroinvertebrate communities [113]. Bispo et al. [77] and Huamantinco and Nessimian [114] indicate that seasonality in rainfall distribution is an important factor in determining the diversity of invertebrates in streams. Differences in morphology and behavioral and physiological traits of species along climatic gradients are common [115,116]. Regions with similar climatic characteristics should favor organisms with similar ecological characteristics, whereas regions under different climate should favor organisms with different characteristics [115,116]. Consequently, morphological traits and species composition must change with climatic changes [117,118]. However, at large spatial scales, Odonata variability may be related to distinct precipitation patterns, such as our study area (1586 to 2269 mm/year). The characteristics of aquatic environments (e.g., physicochemical variables of water) can be influenced by the runoff of precipitation throughout the local drainage basin, consequently affecting species composition.

The number of species, and their frequency of occurrence may decrease with increasing altitude, mainly due to the high temperature variation associated with height [119]. However, the relationship between species richness and altitude is not observed between areas with small altitudinal differences [120], such as our study area (4 to 163 m). Therefore, the altitudinal differences are possibly too small among the streams sampled in this study and therefore may have had no effect on the adult Odonata community.

In general, the Odonata composition may be influenced by regional (e.g., water limitation, temperature, and environmental heterogeneity; [121]) and local factors (e.g., physical and chemical characteristics of the water, canopy cover, and human impact; [41,42,49,69]). This corroborates with studies in neotropical regions which reported that the local factors (e.g., width, depth, canopy, flow, and cover) as well as factors operating on a larger scale (e.g., altitude and precipitation) can explain the variability in benthic communities structuring [104].

In this context, considering the accelerated climatic changes, the scale in which habitat is measured becomes a relevant matter to understand community structure. To understand how communities vary across landscapes or geographic regions (assessing species composition among communities, spatial heterogeneity within and between communities) and the degree of ecological affinity between species and environmental factors (local and regional) is of extreme importance for an effective assessment of diversity maintenance.

## 5. Conclusions

The effects of local and regional abiotic factors interact in structuring Odonata communities. The relationship between the Odonata community and habitat characteristics identified in this study has important implications, as the use of Odonata for monitoring in small streams of the Eastern Amazon is considered. The contrasting responses of the two Odonata suborders (abundance, species richness, and composition) related to the environmental gradient, provide a reliable tool for assessing aquatic habitat changes. Understanding these patterns is a crucial tool to plan long-term strategies for the conservation of aquatic biodiversity. Thus, the recovery of aquatic ecosystems becomes a priority to restore local conditions (restoring them to the landscape), mainly through reconstruction of the physical conditions (e.g., reforestation) and adjustment of the ecological conditions of the waterbodies. Finally, we recommend considering different scales (local and regional) in future studies, since an adequate habitat must meet the ecological needs of all the life stages of these individuals.

## Figures and Tables

**Table 1:** Variance partitioning (with associated *p* Values) using distance matrix of total Odonata, Anisoptera, and Zygoptera species, in two regions of Eastern Amazon, Pará, Brazil. [LO] Pure local abiotic factors; [RE] Pure regional abiotic factors; [RE:LO] variance component shared by regional and local abiotic factors; [RS] variance that is not explained by either local or regional abiotic factors. Adjusted coefficient of determination (Adj. *R*^2^).

Order/Separate Suborders	Variance Partitioning	
	Adj. *R*^2^	*p*
**Odonata**		
Local [LO]	0.046	0.001
Regional [RE]	0.063	0.001
Shared [RE:LO]	0.100	
Residual [RS]	0.790	
**Anisoptera**		
Local [LO]	0.037	0.002
Regional [RE]	0.040	0.001
Shared [RE:LO]	0.084	
Residual [RS]	0.837	
**Zygoptera**		
Local [LO]	0.022	0.001
Regional [RE]	0.069	0.001
Shared [RE:LO]	0.093	
Residual [RS]	0.813	

## Supplementary Materials

The following are available online at https://www.mdpi.com/2075-4450/10/12/446/s1, Table S1: Values obtained for the environmental variables used (regional and local), by stream (ST) sampled in two regions (Santarém—STM and Paragominas—PGM) of the Eastern Amazon, Pará, Brazil: Local: HII (habitat integrity index); WAT (water temperature °C); ECO (electrical conductivity µS/cm); DIO (dissolved oxygen mg/L); pH (hydrogen potential); CAC (canopy cover%); Regional: FOC (% primary forest at catchment buffer scale); FOR (% primary forest at riparian network 100m buffer scale); ALT (altitude m); BIO1 (annual mean temperature); BIO12 (annual precipitation); BIO15 (precipitation seasonality—coefficient of variation). Table S2: List of Odonata (Insecta) species sampled by type of environment (preserved- PRE and altered-ALT) in two regions (Santarém and Paragominas) of the Eastern Amazon, Pará, Brazil.
