# Supplementary material for: The Response of Neotropical Dragonflies (Insecta: Odonata) to Local and Regional Abiotic Factors in Small Streams of the Amazon
Source: Insects. 2019 Dec 12;10(12):446. doi: 10.3390/insects10120446 (PMC6956063; doi:10.3390/insects10120446)
Supplement: Supplementary file 1 [file insects-10-00446-s001.pdf]

Table S1. Values obtained for the environmental variables used (regional and local), by stream (ST) sampled in two regions (Santarém-STM and Paragominas-PGM) of the Eastern Amazon, Pará, Brazil: **Local:** HII (Habitat Integrity Index); WAT (water temperature ºC); ECO (electrical conductivity µS/cm); DIO (dissolved oxygen mg/L); pH (hydrogen potential); CAC (canopy cover %); **Regional:** FOC (% primary forest at catchment buffer scale); FOR (% primary forest at riparian network 100m buffer scale); ALT (altitude m); BIO1 (annual mean temperature); BIO12 (annual precipitation); BIO15 (precipitation seasonality - coefficient of variation).

Stream code/ Municipalities	Environmental variables											
	Local						Regional					
	HII	WAT	ECO	DIO	pH	CAC	FOC	FOR	ALT	BIO1	BIO12	BIO15
**ST1/STM**	0.68	24.8	13.0	7.1	4.09	97.05	21.70	0.00	137	25.3	2055	68
**ST2/STM**	0.49	25.5	16.0	6.57	4.12	96.79	4.80	26.99	135	25.3	2041	68
**ST3/STM**	0.57	25.9	14.6	7.1	5.16	92.78	22.08	9.11	94	25.4	2127	69
**ST4/STM**	0.29	25.4	11.0	7.52	3.8	20.05	37.79	26.41	92	25.4	2184	70
**ST5/STM**	0.96	25.5	27.0	5.8	1.48	98.39	56.85	39.22	135	25.3	1977	67
**ST6/STM**	0.94	27.6	19.0	6.7	2.48	98.12	38.20	21.70	116	25.4	1973	66
**ST7/STM**	0.81	24.9	22.0	5.4	6.3	94.38	99.70	100.00	59	25.7	2010	65
**ST8/STM**	0.90	24.6	23.0	6.63	5.93	98.52	98.78	100.00	68	25.6	2003	65
**ST9/STM**	0.89	24.8	20.0	6.7	4.12	96.12	99.25	99.45	52	25.8	2007	65
**ST10/STM**	0.29	25.1	14.0	5.0	3.08	93.85	64.31	51.72	58	25.6	2104	68
**ST11/STM**	0.81	25.0	13.6	4.9	4.99	99.06	11.25	9.71	59	25.6	2102	68
**ST12/STM**	0.81	25.5	15.2	5.0	4.86	98.52	15.23	11.48	63	25.6	2129	69
**ST13/STM**	0.85	24.7	21.0	7.04	4.15	92.37	66.53	95.55	78	25.6	2065	67
**ST14/STM**	0.29	27.4	7.0	8.1	4.3	41.97	84.27	85.18	75	25.6	2012	65
**ST15/STM**	0.87	25.6	20.6	5.04	4.6	95.85	72.60	72.85	102	25.6	1983	64
**ST16/STM**	0.54	29.67	20.0	5.45	5.4	99.33	29.01	0.40	66	25.7	1993	65
**ST17/STM**	0.81	23.75	10.0	7.4	3.25	96.39	99.50	97.90	139	25.5	1951	63
**ST18/STM**	0.89	25.3	12.0	4.4	3.54	95.58	99.67	98.66	126	25.5	1961	63
**ST19/STM**	0.92	24.3	15.16	5.44	4.88	95.05	79.69	69.42	97	25.6	1943	64
**ST20/STM**	0.97	24.6	16.4	6.7	4.8	98.79	99.96	100.00	100	25.6	1946	64
**ST21/STM**	0.59	25.4	15.0	7.0	3.8	89.57	99.53	98.82	104	25.6	1937	64
**ST22/STM**	0.75	24.4	18.0	6.1	4.74	97.99	54.60	65.91	127	25.6	1885	62
**ST23/STM**	0.15	28.53	17.0	7.45	0.0	14.57	53.07	48.87	96	25.7	1893	62
**ST24/STM**	0.71	29.9	14.0	7.3	3.53	71.92	72.04	61.93	100	25.7	1893	62

**ST25/STM**	0.89	23.5	15.0	6.7	5.5	91.44	100.00	100.00	146	25.5	1880	62
**ST26/STM**	0.89	24.8	14.63	6.83	4.61	97.99	78.23	72.64	117	25.8	1837	61
**ST27/STM**	0.52	25.4	16.0	5.2	2.05	95.98	71.59	72.58	96	25.9	1852	62
**ST28/STM**	0.44	24.8	13.0	7.04	4.0	92.24	78.06	67.18	131	25.7	1851	61
**ST29/STM**	0.55	25.1	13.38	4.01	5.18	97.45	20.98	16.24	95	25.5	2076	68
**ST30/STM**	0.22	27.7	17.0	7.6	5.1	10.96	56.91	37.65	101	25.7	1866	62
**ST31/STM**	0.59	25.22	17.0	5.11	4.8	93.44	14.72	11.23	75	25.5	2182	70
**ST32/STM**	0.54	25.72	22.0	4.63	4.55	35.82	21.48	8.65	81	25.6	2141	69
**ST33/STM**	0.76	26.4	23.0	3.52	4.93	51.33	15.86	11.34	81	25.5	2196	70
**ST34/STM**	0.28	25.0	21.0	3.22	4.65	94.51	73.48	85.49	31	25.8	2037	67
**ST35/STM**	0.99	25.3	16.0	6.45	4.82	97.45	55.34	18.22	115	25.4	1982	66
**ST36/STM**	0.60	24.23	15.0	6.63	4.7	97.99	28.67	15.13	98	25.4	2063	68
**ST37/STM**	0.68	26.34	23.0	5.7	4.35	95.85	5.63	0.00	89	25.6	2108	69
**ST38/STM**	0.40	25.9	19.0	6.7	7.4	61.76	52.85	46.61	121	25.6	1909	62
**ST39/STM**	0.35	25.41	22.0	5.75	4.45	99.06	29.72	39.93	153	25.3	1950	66
**ST40/STM**	0.69	25.86	21.0	7.54	2.11	49.33	14.42	15.06	49	25.8	2083	68
**ST41/STM**	0.81	25.0	18.0	5.55	4.0	99.33	72.18	81.84	4	26.0	2053	67
**ST42/STM**	0.56	25.06	15.0	5.7	4.3	94.65	87.41	80.76	78	25.6	2024	65
**ST43/STM**	0.57	23.5	15.08	4.45	5.0	72.45	74.14	63.04	62	25.7	2008	66
**ST44/STM**	0.62	26.77	15.5	6.11	4.65	58.55	84.82	77.04	51	25.9	1934	64
**ST45/STM**	0.88	23.97	17.0	7.3	2.56	91.84	99.51	100.00	120	25.5	1930	63
**ST46/STM**	0.62	25.2	14.51	6.11	4.09	64.03	83.82	72.23	102	25.7	1907	63
**ST47/STM**	0.46	24.9	17.0	6.7	4.23	96.65	84.23	65.65	57	26.0	1916	63
**ST48/STM**	0.94	24.8	21.0	6.2	3.9	95.58	89.93	74.41	57	26.0	1915	62
**ST49/PGM**	0.51	25.5	18.4	3.9	5.7	87.43	41.45	21.02	41	26.8	2182	75
**ST50/PGM**	0.73	24.9	24.0	3.05	7.7	81.68	65.51	25.17	55	26.8	2182	75
**ST51/PGM**	0.55	26.0	22.4	4.59	4.57	79.54	52.52	49.21	46	26.8	1996	79
**ST52/PGM**	0.53	24.6	27.0	3.66	4.2	68.04	78.78	52.35	52	26.8	1991	80
**ST53/PGM**	0.47	28.2	26.0	4.7	5.84	40.1	1.85	1.50	53	26.8	1977	80
**ST54/PGM**	0.59	24.2	19.7	4.7	6.6	88.63	56.95	33.90	75	26.7	1868	73
**ST55/PGM**	0.71	24.5	39.1	6.02	5.83	93.71	59.01	41.94	84	26.7	1774	78
**ST56/PGM**	0.64	24.66	37.74	5.65	4.31	88.5	68.28	62.95	73	26.8	1803	85
**ST57/PGM**	0.76	24.5	34.3	4.5	4.11	95.45	90.55	89.06	95	26.6	1781	85

**ST58/PGM**	0.64	24.2	44.6	3.93	7.75	89.97	78.44	53.37	110	26.6	1661	84
**ST59/PGM**	0.52	29.1	37.7	2.96	7.09	36.89	46.00	24.18	83	26.7	1642	85
**ST60/PGM**	0.34	25.6	29.4	4.4	4.64	38.63	67.59	67.56	91	26.7	1837	86
**ST61/PGM**	0.49	28.1	35.5	6.76	4.64	41.17	46.84	52.43	104	26.6	1613	89
**ST62/PGM**	0.70	24.8	46.9	4.06	4.78	75.93	46.86	37.90	104	26.6	1604	89
**ST63/PGM**	0.65	24.6	53.8	1.96	4.81	88.36	71.29	77.78	104	26.6	1604	89
**ST64/PGM**	0.47	26.4	26.6	2.44	5.64	44.11	69.28	76.52	114	26.3	2011	68
**ST65/PGM**	0.66	26.3	28.1	3.84	4.75	70.72	69.14	56.94	103	26.4	2006	69
**ST66/PGM**	0.96	24.2	19.01	3.58	3.02	92.05	100.00	100.00	99	26.9	2109	84
**ST67/PGM**	0.88	23.5	19.5	6.15	4.75	99.46	100.00	100.00	109	26.8	2103	84
**ST68/PGM**	0.47	26.9	36.3	3.47	6.02	43.04	36.88	34.12	92	26.6	1769	84
**ST69/PGM**	0.60	27.2	36.7	2.6	5.8	21.39	60.21	61.32	136	26.4	1718	84
**ST70/PGM**	0.72	24.9	33.1	4.91	4.34	4.14	96.09	91.28	126	26.7	1908	86
**ST71/PGM**	0.63	24.6	35.1	3.93	4.94	18.18	31.56	29.21	113	26.7	1884	87
**ST72/PGM**	0.55	24.3	39.1	4.8	4.6	86.76	63.48	66.27	108	26.8	1873	87
**ST73/PGM**	0.53	27.4	40.2	6.5	5.49	90.5	75.26	56.30	89	26.7	1664	84
**ST74/PGM**	0.70	29.2	37.0	4.7	6.5	35.29	48.65	28.69	83	26.7	1626	85
**ST75/PGM**	0.39	22.5	19.7	3.8	5.01	90.77	25.30	8.75	89	26.7	1586	86
**ST76/PGM**	0.70	26.4	19.0	3.9	4.91	84.17	81.66	52.40	116	26.6	1791	87
**ST77/PGM**	0.57	24.6	19.8	4.7	6.48	94.38	81.48	79.61	77	26.8	1860	85
**ST78/PGM**	0.71	24.6	19.01	4.81	6.66	88.23	89.93	91.21	95	26.4	1973	71
**ST79/PGM**	0.52	24.5	17.5	5.5	5.4	96.92	62.20	59.21	85	26.7	1760	86
**ST80/PGM**	0.77	24.4	19.0	4.28	6.56	86.82	97.70	93.12	71	26.9	2024	83
**ST81/PGM**	0.75	24.4	19.42	2.6	4.7	96.65	98.80	98.42	76	26.8	1988	84
**ST82/PGM**	0.47	24.6	20.8	4.6	5.17	94.65	4.49	7.11	69	26.7	1802	78
**ST83/PGM**	0.47	27.7	22.4	4.6	6.31	76.87	68.49	43.89	67	26.7	1871	73
**ST84/PGM**	0.38	25.5	21.4	3.5	6.19	29.94	6.07	4.07	86	26.6	1862	74
**ST85/PGM**	0.46	23.9	47.3	4.41	7.0	95.05	74.50	70.20	120	26.4	1810	77
**ST86/PGM**	0.96	25.0	76.2	4.3	4.61	98.12	95.98	98.41	163	26.2	1780	78
**ST87/PGM**	0.87	27.1	55.4	5.07	5.06	2.67	97.47	100.00	148	26.4	1721	80
**ST88/PGM**	0.62	25.74	41.55	5.97	4.01	86.49	69.30	57.31	65	26.8	1746	86
**ST89/PGM**	0.46	25.2	28.3	6.36	4.1	40.24	28.54	11.34	66	26.7	2139	75
**ST90/PGM**	0.38	24.6	33.0	4.07	4.42	97.19	26.99	41.67	88	26.7	1710	87

**ST91/PGM**	0.76	26.1	21.1	3.0	6.12	58.28	99.60	100.00	89	26.9	2025	85
**ST92/PGM**	0.47	24.4	20.9	4.2	6.47	96.92	82.17	71.90	107	26.5	1798	79
**ST93/PGM**	0.85	25.0	33.3	4.33	5.64	88.1	99.91	100.00	113	26.8	2122	84
**ST94/PGM**	0.78	23.7	24.0	3.37	6.21	47.45	99.40	100.00	112	26.8	2065	84
**ST95/PGM**	0.86	26.7	21.85	5.7	4.4	81.28	99.14	99.41	126	26.7	1994	85
**ST96/PGM**	0.28	26.3	51.0	5.25	5.7	5.88	73.51	45.97	73	26.7	1760	80
**ST97/PGM**	0.85	24.7	35.4	6.15	5.5	94.38	98.43	99.28	87	27.0	2269	81
**ST98/PGM**	0.88	24.7	65.0	6.19	4.81	94.51	59.25	89.07	111	26.5	1768	80

Table S2. List of Odonata (Insecta) species sampled by type of environment (preserved- PRE and altered-ALT) in two regions (Santarém and Paragominas) of the Eastern Amazon, Pará, Brazil.

Suborders / Families / Species	Santarém			Paragominas			General total
	PRE	ALT	Total	PRE	ALT	Total	
**ANISOPTERA**							
**Libellulidae**							
Argyrothemis argentea Ris, 1909	4	1	**5**	5	16	**21**	**26**
Dasythemis esmeralda Ris, 1910	1	7	**8**	0	1	**1**	**9**
Diastatops obscura (Fabricius, 1775)	3	6	**9**	5	100	**105**	**114**
Dythemis multipunctata multipunctata Kirby, 1894	0	0	**0**	0	1	**1**	**1**
Elasmothemis cannacrioides (Calvert, 1906)	0	3	**3**	0	8	**8**	**11**
Elasmothemis williamsoni (Ris, 1919)	0	1	**1**	0	0	**0**	**1**
Erythemis credula (Hagen, 1861)	0	0	**0**	0	2	**2**	**2**
Erythemis haematogastra (Burmeister, 1839)	0	4	**4**	0	3	**3**	**7**
Erythemis sp.	0	0	**0**	0	1	**1**	**1**
Erythemis vesiculosa (Fabricius, 1775)	0	6	**6**	0	0	**0**	**6**
Erythrodiplax amazonica Sjöstedt, 1918	0	0	**0**	3	21	**24**	**24**
Erythrodiplax avittata Borror, 1942	0	2	**2**	0	0	**0**	**2**
Erythrodiplax basalis (Kirby, 1897)	3	56	**59**	11	238	**249**	**308**
Erythrodiplax castanea (Burmeister, 1839)	0	2	**2**	0	0	**0**	**2**
Erythrodiplax fusca (Rambur, 1842)	15	96	**111**	1	95	**96**	**207**
Erythrodiplax juliana Ris, 1911	0	7	**7**	0	5	**5**	**12**
Erythrodiplax melanica Borror, 1942	0	1	**1**	0	0	**0**	**1**
Erythrodiplax nigricans (Rambur, 1842)	2	6	**8**	0	0	**0**	**8**
Erytthrodiplax ochracea (Burmeister, 1839)	0	0	**0**	0	1	**1**	**1**
Erythrodiplax paraguayensis (Förster, 1905)	0	3	**3**	0	0	**0**	**3**
Fylgia amazonica amazonica Kirby, 1889	2	1	**3**	3	0	**3**	**6**
Gynothemis sp.	0	0	**0**	1	1	**2**	**2**
Macrothemis sp.	0	0	**0**	0	6	**6**	**6**
Macrothemis absimilis Costa, 1991	0	1	**1**	0	0	**0**	**1**
Macrothemis ludia Belle, 1987	0	0	**0**	0	1	**1**	**1**
Miathyria marcella (Selys *in* Sagra, 1857)	0	0	**0**	0	1	**1**	**1**
Miathyria simplex (Rambur, 1842)	0	0	**0**	0	3	**3**	**3**
Micrathyria aequalis (Hagen, 1861)	1	1	**2**	0	0	**0**	**2**
Micrathyria artemis Ris, 1911	2	3	**5**	0	7	**7**	**12**
Micrathyria eximia Kirby, 1897	0	0	**0**	0	1	**1**	**1**
Micrathyria hesperis Ris, 1911	0	4	**4**	0	0	**0**	**4**
Micrathyria pseudeximia Westfall, 1992	0	0	**0**	0	1	**1**	**1**
Micrathyria romani Sjöstedt, 1918	3	6	**9**	0	0	**0**	**9**
Micrathyria sp.1	0	0	**0**	0	3	**3**	**3**
Micrathyria sp.2	0	0	**0**	0	1	**1**	**1**
Micrathyria ungulata Förster, 1907	0	2	**2**	0	0	**0**	**2**
Nephepeltia berlai Santos, 1950	1	0	**1**	0	0	**0**	**1**
Nephepeltia flavifrons (Karsch, 1889)	0	1	**1**	0	0	**0**	**1**
Oligoclada abbreviata abbreviata (Rambur, 1842)	0	0	**0**	16	18	**34**	**34**
Oligoclada amphinome Ris, 1919	21	0	**21**	0	0	**0**	**21**
Oligoclada crocogaster Borror, 1931	0	0	**0**	0	4	**4**	**4**
Oligoclada raineyi Ris, 1919	0	0	**0**	0	24	**24**	**24**
Oligoclada stenoptera Borror, 1931	2	1	**3**	0	0	**0**	**3**
Oligoclada walkeri Geijskes, 1931	3	0	**3**	49	25	**74**	**77**
Oligoclada xanthopleura Borror, 1931	0	4	**4**	0	0	**0**	**4**
Orthemis biolleyi Calvert, 1906	0	0	**0**	1	2	**3**	**3**

Orthemis discolor (Burmeister, 1839)	5	30	**35**	0	25	**25**	**60**
Perithemis cornelia Ris, 1910	0	0	**0**	1	2	**3**	**3**
Perithemis lais (Perty, 1834)	15	9	**24**	9	28	**37**	**61**
Perithemis sp.1	0	0	**0**	0	1	**1**	**1**
Perithemis sp.2	0	0	**0**	1	1	**2**	**2**
Rhodopygia ccardinalis (Erichson *in* Schomburgk, 1848)	0	0	**0**	0	9	**9**	**9**
Zenithoptera fasciata (Linnaeus, 1758)	0	2	**2**	0	0	**0**	**2**
Zenithoptera lanei Santos, 1941	0	9	**9**	2	31	**33**	**42**
Zenithoptera viola Ris, 1910	0	0	**0**	0	1	**1**	**1**
**Gomphidae**							
Cacoides latro (Erichson *in* Schomburgk, 1848)	0	0	**0**	0	2	**2**	**2**
Phyllocycla bartica Calvert, 1948	1	2	**3**	0	0	**0**	**3**
Phyllogomphoides cepheus Belle, 1980	0	0	**0**	1	0	**1**	**1**
Progomphus intricatus Hagen *in* Selys, 1858	0	1	**1**	0	0	**0**	**1**
Progomphus maculatus Belle, 1984	1	0	**1**	0	0	**0**	**1**
Progomphus sp.	0	0	**0**	0	3	**3**	**3**
Zonophora calippus calippus Selys, 1869	1	1	**2**	0	0	**0**	**2**
**Aeshnidae**							
Gynacantha membranalis Karsch, 1891	0	0	**0**	6	0	**6**	**6**
**ZYGOPTERA**							
**Coenagrionidae**							
Acanthagrion adustum Williamson, 1916	0	0	**0**	0	26	**26**	**26**
Acanthagrion aepiolum Tennessen, 2004	0	0	**0**	2	4	**6**	**6**
Acanthagrion apicale Selys, 1876	0	2	**2**	3	5	**8**	**10**
Acanthagrion ascendens Calvert, 1909	0	0	**0**	3	4	**7**	**7**
Acanthagrion chicomendesi Machado, 2012	0	0	**0**	1	0	**1**	**1**
Acanthagrion jessei Leonard, 1977	0	0	**0**	0	1	**1**	**1**
Acanthagrion kennedii Williamson, 1916	1	1	**2**	3	16	**19**	**21**
Acanthagrion rubrifrons Leonard, 1977	0	0	**0**	3	0	**3**	**3**
Acanthagrion sp.	0	0	**0**	0	1	**1**	**1**
Acanthallagma luteum Williamson & Williamson, 1924	0	0	**0**	3	8	**11**	**11**
Argia chapadae Calvert, 1909	0	3	**3**	0	0	**0**	**3**
Argia eliptica Selys, 1865	10	8	**18**	0	0	**0**	**18**
Argia euphorbia Fraser, 1946	7	1	**8**	0	0	**0**	**8**
Argia fumigata Hagen *in* Selys, 1865	0	9	**9**	2	1	**3**	**12**
Argia infumata Selys, 1865	45	1	**46**	54	0	**54**	**100**
Argia insipida Hagen *in* Selys, 1865	0	0	**0**	2	0	**2**	**2**
Argia mollis Hagen *in* Selys, 1865	0	0	**0**	1	3	**4**	**4**
Argia reclusa Selys, 1865	0	0	**0**	1	0	**1**	**1**
Argia smithiana Calvert, 1909	0	0	**0**	11	0	**11**	**11**
Argia sp.	24	29	**53**	0	0	**0**	**53**
Argia sp.1	0	0	**0**	15	1	**16**	**16**
Argia sp.2	0	0	**0**	0	1	**1**	**1**
Argia sp.3	0	0	**0**	22	14	**36**	**36**
Argia sp.4	0	0	**0**	1	0	**1**	**1**
Argia sp.5	0	0	**0**	2	2	**4**	**4**
Argia sp.6	0	0	**0**	0	1	**1**	**1**
Argia sp.7	0	0	**0**	2	0	**2**	**2**
Argia sp.8	0	0	**0**	2	2	**4**	**4**
Argia thespis Hagen *in* Selys, 1865	0	0	**0**	31	13	**44**	**44**
Argia tinctipennis Selys, 1865	47	24	**71**	33	11	**44**	**115**
Argia tupi Calvert, 1909	0	0	**0**	2	0	**2**	**2**

Epipleoneura capilliformis (Selys, 1886)	67	56	**123**	4	0	**4**	**127**
Epipleoneura fuscaenea Williamson, 1915	0	0	**0**	0	5	**5**	**5**
Epipleoneura haroldoi Santos, 1964	25	9	**34**	0	0	**0**	**34**
Epipleoneura metallica Rácenis, 1955	0	0	**0**	51	107	**158**	**158**
Epipleoneura pereirai Machado, 1964	2	0	**2**	0	0	**0**	**2**
Epipleoneura spatulata Rácenis, 1960	0	17	**17**	0	0	**0**	**17**
Epipleoneura westfalli Machado, 1986	0	0	**0**	5	3	**8**	**8**
Ischnura capreolus (Hagen, 1861)	0	0	**0**	0	1	**1**	**1**
Mecistogaster linearis linearis (Fabricius, 1776)	1	0	**1**	1	0	**1**	**2**
Neoneura denticulata Williamson, 1917	0	0	**0**	4	5	**9**	**9**
Neoneura gaida Rácenis, 1953	0	0	**0**	0	1	**1**	**1**
Neoneura joana Williamson, 1917	0	0	**0**	0	2	**2**	**2**
Neoneura luzmarina De Marmels, 1989	6	26	**32**	16	9	**25**	**57**
Neoneura rubriventris Selys, 1860	0	0	**0**	1	22	**23**	**23**
Phasmoneura exigua (Selys, 1886)	5	35	**40**	0	0	**0**	**40**
Protoneura tenuis Selys, 1860	2	0	**2**	61	0	**61**	**63**
Psaironeura tenuissima (Selys, 1886)	25	16	**41**	10	0	**10**	**51**
Telebasis sp.	1	0	**1**	0	0	**0**	**1**
Telebasis sanguinalis Calvert, 1909	0	0	**0**	0	3	**3**	**3**
Tigriagrion aurantinigrum Calvert, 1909	0	0	**0**	4	22	**26**	**26**
**Dicteriadidae**							
Dicterias atrosanguinea Selys, 1853	22	10	**32**	2	0	**2**	**34**
Heliocharis amazona Selys, 1853	12	2	**14**	4	3	**7**	**21**
**Polythoridae**							
Chalcopteryx radians Ris, 1914	61	36	**97**	0	0	**0**	**97**
Chalcopteryx rutilans (Rambur, 1842)	61	55	**116**	30	0	**30**	**146**
**Calopterygidae**							
Hetaerina auripennis (Burmeister, 1839)	0	0	**0**	6	32	**38**	**38**
Hetaerina indeprensa Garrison, 1990	30	97	**127**	0	0	**0**	**127**
Hetaerina rosea Selys, 1853	1	0	**1**	0	0	**0**	**1**
Hetaerina sanguinea Selys, 1853	0	6	**6**	0	4	**4**	**10**
Mnesarete aenea (Selys, 1853)	149	81	**230**	56	4	**60**	**290**
Mnesarete cupraea (Selys, 1853)	0	0	**0**	1	0	**1**	**1**
Mnesarete smaragdina (Selys, 1869)	32	109	**141**	0	0	**0**	**141**
Mnesarete williamsoni Garrison, 2006	0	0	**0**	19	69	**88**	**88**
**Perilestidae**							
Perilestes attenuatus Selys, 1886	0	1	**1**	0	0	**0**	**1**
Perilestes kahli Williamson & Williamson, 1924	0	0	**0**	12	0	**12**	**12**
Perilestes solutus Williamson & Williamson, 1924	0	0	**0**	2	0	**2**	**2**
**Megapodagrionidae**							
Heteragrion aurantiacum Selys, 1862	0	0	**0**	51	1	**52**	**52**
Heteragrion icterops Selys, 1862	3	4	**7**	3	8	**11**	**18**
Heteragrion sp.	119	32	**151**	1	0	**1**	**152**
Oxystigma petiolatum (Selys, 1862)	0	0	**0**	3	0	**3**	**3**
Oxystigma williamsoni Geijskes, 1976	12	14	**26**	0	0	**0**	**26**
**Total**	**856**	**963**	**1819**	**661**	**1108**	**1769**	**3588**
